# A realist synthesis of randomised control trials involving use of community health workers for delivering child health interventions in low and middle income countries

**DOI:** 10.1186/1472-6963-10-286

**Published:** 2010-10-13

**Authors:** Sumit S Kane, Barend Gerretsen, Robert Scherpbier, Mario Dal Poz, Marjolein Dieleman

**Affiliations:** 1 Department of Development, Policy & Practice, Royal Tropical Institute, Mauritskade 63, Amsterdam, The Netherlands; 2 Department of Child and Adolescent Health and Development, World Health Organization, Avenue Appia 20, 1211 Geneva 27, Switzerland; 3 Department of Human Resources for Health, World Health Organization, Avenue Appia 20, 1211 Geneva 27, Switzerland

## Abstract

**Background:**

A key constraint to saturating coverage of interventions for reducing the burden of childhood illnesses in Low and Middle Income Countries (LMIC) is the lack of human resources. Community health workers (CHW) are potentially important actors in bridging this gap. Evidence exists on effectiveness of CHW in management of some childhood illnesses (IMCI). However, we need to know how and when this comes to be. We examine evidence from randomized control trials (RCT) on CHW interventions in IMCI in LMIC from a realist perspective with the aim to see if they can yield insight into the working of the interventions, when examined from a different perspective.

**Methods:**

The realist approach involves educing the mechanisms through which an intervention produced an outcome in a particular context. 'Mechanisms' are reactions, triggered by the interaction of the intervention and a certain context, which lead to change. These are often only implicit and are actually hypothesized by the reviewer. This review is limited to unravelling these from the RCTs; it is thus a hypothesis generating exercise.

**Results:**

Interventions to improve CHW performance included 'Skills based training of CHW', 'Supervision and referral support from public health services', 'Positioning of CHW in the community'. When interventions were applied in context of CHW programs embedded in local health services, with beneficiaries who valued services and had unmet needs, the interventions worked if following mechanisms were triggered: anticipation of being valued by the community; perception of improvement in social status; sense of relatedness with beneficiaries and public services; increase in self esteem; sense of self efficacy and enactive mastery of tasks; sense of credibility, legitimacy and assurance that there was a system for back-up support. Studies also showed that if context differed, even with similar interventions, negative mechanisms could be triggered, compromising CHW performance.

**Conclusion:**

The aim of this review was to explore if RCTs could yield insight into the working of the interventions, when examined from a different, a realist perspective. We found that RCTs did yield some insight, but the hypotheses generated were very general and not well refined. These hypotheses need to be tested and refined in further studies.

## Background

Around 9.7 million children die every year, almost all in Low and Middle Income Countries (LMIC) [1]. More than 60% of these deaths are preventable if existing interventions can be made available universally [2]. Many of these interventions can be delivered at the community level [2]. A key constraint in saturating the coverage of these interventions is the lack of human resources [3]. Currently, there is a renewed interest in Community health workers (CHW) as potential important actors in bridging the human resource gap and in improving the reach of health services [3-7], particularly towards achieving child mortality related millennium development goals in LMIC. World Health Organisation's (WHO) multi-country evaluation of Integrated Management of Childhood Illnesses (IMCI) implementation also concluded that it was necessary to expand health care delivery systems to include community based interventions [8].

A number of reviews on the subject have tried to examine evidence to improve the operationalization of interventions by CHWs, including for child health. Lehmann et al [9] and Lewin et al [10] have reviewed evidence on CHW interventions in LMIC and Haines et al [11] have particularly so for child health. Lewin et al [10] found lay health workers to be effective in specific areas in child health, when compared to usual care. Haines et al [11] highlight the contextual nature of CHW's performance. Both caution that CHW interventions are not the panacea for all that ails the health systems in LMIC and that large scale CHW programmes should be initiated with great caution. Both raise questions about the applicability of findings to different settings and about the conditions under which CHW interventions should be implemented. Haines et al [11] and Bhattacharya et al [12] found that both monetary and non monetary incentives are important for CHW's performance. Lehmann et al [9] take a historical perspective on the operational challenges for CHW interventions, they articulate the tenuous positioning of CHWs vis-à-vis their roles and expectations, within the health system and the communities they serve. They, like Victora et al [13] found that success (or failure) of CHW interventions and the performance of CHWs is contingent upon a range of contextual factors. Indeed, CHW interventions are complex interventions embedded within complex health and social systems and contexts.

A realist perspective can help gain insight into the context within which complex interventions achieved results elsewhere and can give an understanding of the mechanisms that led to it [14]. Using the realist perspective for examining and reviewing existing evidence is a relatively new method in public health. Few such exercises have been done, fewer still published [15-18]. Dieleman et al [18] reviewed 48 articles reporting the results of human resource management interventions in LMIC for improving health worker performance. They used a realist perspective to explore why some interventions worked in certain contexts and not in some. Their review revealed a large set of potential mechanisms for improving health worker performance that could be triggered by the interaction between the intervention and context. Their findings seemed to corroborate findings from earlier studies regarding the mechanisms that could lead to better health worker performance. Their review demonstrates the complexities associated with reviewing evidence from a variety of sources and focused on different levels of effect. Greenhalgh et al's [15] conducted a realist review of trials involving school feeding programmes. Their review was an attempt to see existing evidence from a realist perspective in order to gain insight into how these interventions might have produced their outcomes. In our view, they struggled to come up with a credible and plausible set of mechanisms and hypotheses. Because their enquiry was limited to trials and to trials in very different contexts, they were also not able to sufficiently test these hypotheses. However, van der Knaap et al [17] have argued that findings from realist synthesis can be generalisable if the evidence used for the synthesis is more internally valid. Since, randomised control trials (RCTs) have high internal validity, in this paper we review RCTs of interventions involving CHWs for improving child health in LMIC from a realist perspective with the aim to see if the RCTs can yield insight into the working of the CHWs. RCTs involving CHW interventions, when examined from a realist perspective, can yield generic hypotheses about what works, for whom, and in which circumstances. These hypotheses can then be refined through further literature reviews and can be tested empirically. We believe that we would be the first to articulate hypotheses about CHW performance using a realist approach.

## Methods

### Inclusion criteria

Types of studies: RCT, including cluster RCT (CRCT), of CHW interventions for improving child health, published in peer reviewed journals, in the period 1997 to 2008. Only studies from LMIC were included.

### Types of interventions

Interventions delivered by CHWs; CHW, as referred to by a WHO working group in 1989 [19]:- "Community health workers should be members of the communities where they work, should be selected by the communities, should be answerable to the communities for their activities, should be supported by the health system but not necessarily a part of its organization, and have shorter training than professional workers."

Studies which measured the effectiveness of CHW trainings through pre and post test examinations, interventions where the worker was available only to his/her family and not to the whole community, interventions where CHW's performance could not be isolated from that of other workers and where primary outcomes could not be isolated for children, were excluded. We limited ourselves to interventions targeting children between 1 to 60 months of age.

### Search Strategy

Search conducted in Nov 2008. Keywords used were: Community Health Workers; Community Health Aide; Village Health Worker; Barefoot Doctor; Community Workers; Community based intervention. Limits included: Child, Pre School; Infant; Randomized control trials; Controlled clinical trials; English language. Pubmed, Popline, Embase, CINAHL, COCHRANE and CENTRAL databases were searched.

**Figure 1:**
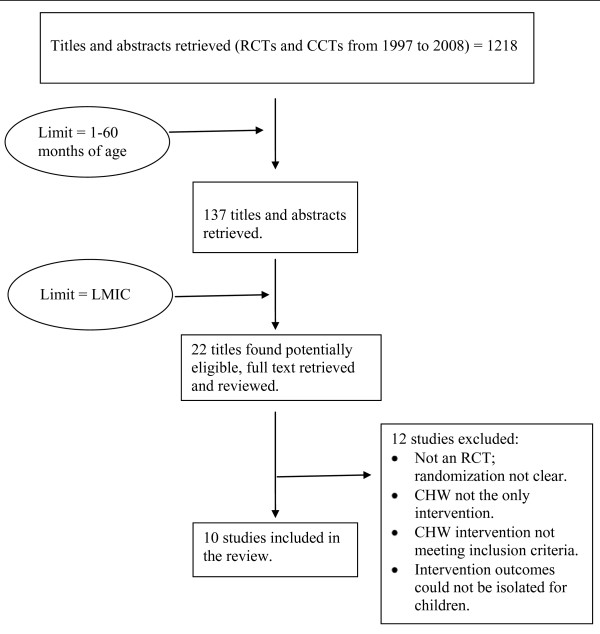
**Search Flow**.

Lewin et al [10] and Haines et al's[11] reviews on the subject were the starting points for this work. Bibliographies of reviews [4,10,11,20] and studies retrieved on the subject were hand searched. Of the 137 titles retrieved, ten RCT/CRCT fulfilled the inclusion criteria (Figure 1: Search Flow). These were independently read by the three authors. The three authors agreed on including these in the review. For the purpose of this review, we define health worker performance along the lines of the world health report 2006 [21], and Dieleman and Harnmeijer [22].

### Theoretical framework: The Realist Approach

Pawson and Tilley [14] and Pawson et al [23,24] have proposed a method for reviewing and synthesizing evidence on complex interventions; it is based on the 'realist' approach. In the 'realist' approach, one examines the interaction between the context and the intervention and attempts to unravel and explain the mechanisms that are set in motion by this interaction to produce the outcomes. This is done through an interpretive, reflexive and iterative process. The term 'mechanisms', as used throughout this work refers to the reactions, triggered by the intervention within a certain context, which lead to a certain outcome. It is the pivot around which realist enquiry revolves. Using a realist approach, in this review we elicit Context Mechanism Outcome Configurations (CMOC) from the RCTs. CMOC are the articulation of the interaction between the intervention, the context within which it is applied and the mechanisms that are set in motion by this interaction - leading up to an outcome. In the realist method these serve as hypotheses that need to be tested and refined.

We chose to review only RCTs because by some measures they are considered the gold standard of evidence and have high internal validity to their outcomes. We chose RCTs also because they are the building blocks of most systematic reviews that policy makers use to inform policies. We reviewed the RCTs using a realist perspective to see if we could identify CMOC from within what was reported by the authors. The mechanisms were included only when they were either researched or discussed by the authors of the RCTs. Over multiple rounds of discussions and argumentation, a common understanding of these findings (CMOC) was arrived at between the three authors. This review is limited to extracting CMOC from the papers under review; it is thus only a hypotheses generating exercise. A broader, extensive realist review of the literature can help reveal the whole spectrum of CMOC. Such a review can then help find a more complete set of CMOC and to test these hypotheses to come up with refined middle range explanatory theories.

## Findings

### Study characteristics

Six RCTs [25-30] and four Cluster RCTs [31-35] were reviewed. Six trials focused on CHW interventions for promoting exclusive breast feeding [27,32,30,33]. One trial each focused on malaria [26], diarrhoea [31] and nutrition [34,35]. One examined CHW's effectiveness for child health interventions in general [25].

### Interventions

In all the RCTs, the intervention involved service delivery at homes of beneficiaries. In all the RCTs, the intervention actually consisted of a bundle of interventions. We identified three broad categories of interventions in the RCTs we reviewed. Training: which involved the provision of knowledge and skills based training to CHW and included practice sessions. Systemic Interventions: This involved establishment of clear roles and specific responsibilities (roles made clear to CHW, other health workers and beneficiary communities); it also included rigorous supervision and mentoring of CHWs by formal health service professionals and good referral support from the formal health service. Interventions involving the positioning of the CHW within the beneficiary community: through the CHW being explicitly selected by beneficiary community and the CHW being positioned as a role model within the beneficiary community.

### Outcomes

The ten trials reported a variety of primary and secondary outcomes. We inductively categorised these outcomes from the point of view of assessing the CHW's performance, as being positive - when the intervention worked or negative. In eight trials, the intervention had a positive outcome and the CHW's performance was good. In one trial the intervention had a negative outcome, but the CHW's performance was good [34,35]. In one trial the intervention had a negative outcome and the CHW's performance was not good [25].

Additional File 1 summarises the RCTs, the context, the intervention, the mechanisms triggered and the reported outcomes. Additional File 1 shows that in all the trials, more than one type of intervention was applied to improve CHWs performance. It also shows that the outcomes are reported not in terms of CHW performance, but rather in terms of the consequences of their performance on specific health outcomes.

### CMO Configurations

In six of the trials, the CHW interventions were implemented in urban areas amongst beneficiary communities who were poor and had an unmet need. The interventions were embedded in or closely linked to local public health care services, and were implemented by locally trusted agencies. In such a context when the intervention involved selection or election of CHW from within the beneficiary community, particularly of such individuals who were trusted and seen as role models by the community - it triggered a sense of relatedness between the CHW and beneficiaries, a sense of responsiveness and responsibility amongst CHWs; it also led to a feeling of being valued by peers for fulfilling the needs of their community. When this was so, the interventions had positive outcomes [27-32].

In the same context, when the intervention involved training CHWs on specific tasks targeted at specific situations, and the training was supplemented by practice sessions and on-job mentoring - it triggered a sense of self efficacy amongst CHWs; the skills building and practice sessions helped CHWs gain enactive mastery on the tasks and triggered a sense of confidence in being able to solve problems.

Similarly, when the intervention involved CHWs being supervised and mentored by local formal health services,- it triggered a sense of credibility of being a part of the system and a sense of assurance both for themselves and for the community, that there was a system of back-up in times of need. When this was so, it contributed to the intervention having positive outcomes.

In Kidane et al's study [26], the CHW interventions were implemented in a rural area amongst beneficiary communities who were poor, had an unmet need and in addition had strong community solidarity in view of past collective adverse experiences. In this case too, the CHW intervention was embedded within local health services and the intervention was composed of three key components similar to the trials implemented in urban areas. In spite of the context being slightly different (rural compared to urban settings), the intervention triggered similar mechanisms as in trials set in urban settings.

In Bhandari et al's study [33], the CHW intervention was implemented in a rural area amongst beneficiary communities who were not so poor and probably had only some unmet needs. Here too, the CHW intervention was embedded within local health services, the intervention was composed of similar components, but only the training component triggered similar mechanisms as mentioned above [33]. Unlike other trials, in Bhandari et al [33], an expectation of appreciation by authority (study team during study period) and possibility of being rewarded was explicitly reported as a factor that may have motivated CHW's to perform better.

In another rural context, with the CHW intervention targeting poor beneficiaries with unmet needs [25], with a similar training intervention, but when the CHW's were not explicitly chosen by beneficiaries and were appointed by the political establishment - it led to an absence of relatedness and responsibility amongst CHWs. It compromised the CHW's motivation to perform, undermining intervention outcomes [25].

In the same context, because the CHW's were not working together with, nor supervised by local health services, it led to poor sense of legitimacy of the CHW amongst beneficiaries and formal health services. When this was so, the CHW's performance and intervention outcomes were compromised.

In the same study, where the CHW intervention was embedded within the local public health services, but the intervention did not clearly articulate the CHW's roles (to CHWs, to beneficiaries and to the health services), the uncertainty because of ill defined roles led to lowered motivation, less involvement (of CHWs, of cadres of formal health services and beneficiaries), and ultimately to poor performance and outcomes [25]. The lack of clarity of roles in the intervention also led to an environment of confusion (for CHWs, cadres of public health services and beneficiaries alike) and compromised the outcomes (performance of CHWs and the intervention outcomes)[25].

When the same intervention elements were applied in a context [35] where the intervention did not address an unmet need of the beneficiaries, it had no value for the beneficiary community. In such a situation, the intervention outcome, in spite of good performance by CHWs, was compromised [35].

### Discussion and Conclusions

The aim of this review was to explore if RCTs could yield insight into the working of interventions involving CHWs for improving child health, when examined from a realist perspective. We found that RCTs did yield some insight, but this insight was very general.

In the *context *of CHW programs targeting the poor with an unmet need, and embedded in or closely linked to local health care services, we can conclude that:

*Training interventions *in the form of knowledge and skills based training complemented by ongoing in-field mentoring, can improve the CHW's performance when they are able to trigger the following *mechanisms:*

• a sense of self efficacy and enactive mastery of the tasks,

• an increase in self esteem,

• assurance that there is a system for back-up support.

*Health system related interventions *in the form of setting clear roles and specific responsibilities for CHWs, ensuring mentoring for CHWs by health workers from local public health services, ensuring good referral support for CHWs from local public health services, can improve the CHW's performance when they are able to trigger the following *mechanisms:*

• a sense of relatedness with the local public health services, and thus accountability towards the system,

• a sense of credibility and legitimacy of being part of the local public health services,

• an anticipation of being valued by the local public health services and the community,

• a perception of improvement in social status,

• an assurance that there is a system for back-up support.

*Interventions involving better positioning of the CHW within communities *(Eg: Selection of the CHWs in consultation with beneficiary communities; the CHWs being members of the beneficiary community, and perceived by them as role models) can improve the CHW's performance when they are able to trigger the following *mechanisms:*

• an anticipation of being valued by the community,

• a perception of improvement in social status, and having a valuable social role

• a sense of relatedness with and accountability to the beneficiaries

This inventory of CMOC, derived from the RCTs under review, is limited, but a beginning nevertheless. This inventory should be seen as a set of generic hypotheses derived from the best existing evidence. The inventory is by no means complete and as stated earlier it is very generic. It is generic because the context has not been sufficiently reported in the RCTs. For example, similar interventions were implemented in urban and rural settings, and they had similar outcomes, but the context (rural vs urban) was not sufficiently disaggregated and described to allow sufficient understanding whether or not different aspects of this broader context triggered different mechanisms. However, we found that many of these generic hypotheses are corroborated by findings from many earlier reviews [4,10,11] giving an inkling of the external validity of these CMOC. For instance, Lehmann and Sanders [4] found, that to be effective, the CHW must not only be from the beneficiary communities, they must conform to the norms and customs of the community they serve. They also found that selection, training, supervision and availability of logistic support are important factors for the CHW's performance; they also found that the CHWs's success is contingent to their embedment in beneficiary communities and support from the government and political establishment. Haines et al [11] point out that CHW interventions can be undermined by a corrupt, partisan and patronage based political establishment. They also point out that CHW intervention outcomes depend on whether power equations in the relationship between CHWs and professionals lead to creation of trust and harmony or rivalry and distrust. Though not labelled as such by the authors, they are in fact referring to mechanisms being triggered by the interaction of the intervention and the context. Haines et al [11] also found that CHWs performed best when they had limited responsibilities and focused tasks. They also found that supportive supervision and support from formal health services is critical to CHW's success. Lewin et al [10] found evidence that lay health workers (CHW) can deliver certain specific services well and not a wide range of services. Bhattacharya et al [12] and Haines et al [11] conclude that to be successful, CHW interventions need to have multiple incentives, simultaneously, at multiple levels (individual, community, and health system levels), tailored to local context. While these reviews do point to the importance of context, we found that most of these reviews gave only limited insight into the context in which various interventions were applied; this was probably due to the focus of the reviews.

During our literature search, we came across a vast array of material on the deployment of the CHWs for child health in LMIC. As a follow up to this review, further realist review of this literature needs be undertaken. This can not only yield a more complete set of CMOC, it can help test and refine these to develop a better understanding of the working of the CHWs in LMIC.

## Limitations and Challenges

We explicitly chose to do a realist review of the RCTs to see what they could additionally yield. While the CHWs were an important component of the interventions being tested in the RCTs, none of the RCTs under review explicitly focused on performance of the CHW as an outcome. The RCTs under review offered a fair amount of information about the interventions, only some information about context - allowing us to formulate only generic hypotheses. Disentangling context from intervention elements was a daunting task, particularly when doing this across RCTs. Trying to unravel the mechanisms through which various component interventions affected the outcome, was even more complex. During our analysis we realized that when intervention components changed or operated differently, it effectively meant that the context for other intervention components had changed, triggering different mechanisms. This made the analysis very complex. We also struggled with distinguishing banal contextual elements from the significant ones.

These struggles are of significance because they serve as pointers to the travails of conducting a full fledged realist review examining all kinds of evidence. Realist reviews can yield valuable insight for policy makers and program planners, but conducting them is a complex and laborious task.

Authors seldom described or discussed the mechanisms that explained their study outcomes. We realise that the RCT design, the exacting reporting requirements and word limits of journals, restrict authors from sharing all their operational experiences. In addition RCTs tend to report average effects and not differential effects of interventions, and less so of the context and rarely of the mechanisms triggered by their interactions. This makes the RCTs less useful for answering the questions regarding how interventions work. These generic hypotheses seem to be recurring in the literature, however they have not been explicitly tested across contexts. We want to make a case for the urgent need for studies (including RCTs) to include greater details on context. If this is done, then future realist reviews can make better use of existing evidence to test and refine such hypotheses and develop context specific program theories. Such context specific program theories will be valuable for guiding the scaling up of public health programmes.

## Competing interests

The authors declare that they have no competing interests.

## Authors' contributions

SK drafted and finalised the manuscript. MD initiated the review. SK, MD, BG reviewed the studies. MD, BG, RS, MP reviewed and commented on the draft manuscripts. All authors have read and approved the final manuscript.

## Pre-publication history

The pre-publication history for this paper can be accessed here:

http://www.biomedcentral.com/1472-6963/10/286/prepub

## Supplementary Material

Additional File 1 **Summaries of the contextual factors identified, the interventions, the mechanisms triggered and the outcomes in the RCTs**. Additional File 1 summarises the RCTs, the context, the intervention, the mechanisms triggered and the reported outcomes. Additional File 1 shows that in all the trials, more than one type of intervention was applied to improve CHWs performance. Click here for file
