# Supplementary material for: A realist synthesis of randomised control trials involving use of community health workers for delivering child health interventions in low and middle income countries
Source: BMC Health Serv Res. 2010 Oct 13;10:286. doi: 10.1186/1472-6963-10-286 (PMC2964693; doi:10.1186/1472-6963-10-286)
Supplement: Additional File 1 — Summaries of the contextual factors identified, the interventions, the mechanisms triggered and the outcomes in the RCTs. Additional File 1 summarises the RCTs, the context, the intervention, the mechanisms triggered and the reported outcomes. Additional File 1 shows that in all the trials, more than one type of intervention was applied to improve CHWs performance. [file 1472-6963-10-286-S1.PDF]

Additional File 1: Summaries of the contextual factors identified, the interventions, the mechanisms triggered and the outcomes in the RCTs.

Authors, Intervention Focus and Area	Design				
		**Context**	**Intervention characteristics**	**Mechanisms triggered**	**Outcomes/Conclusions**
**Exclusive breast feeding**					
Agrasada et al, 2005, Phillipines	RCT<br>N=204	• Urban setting.<br>• Setting - tertiary hospital.<br>• Well established health services.<br>• Beneficiaries were first time mothers with no prior experience of breast feeding.<br>• Beneficiaries were from a social setting with limited or no postnatal support and saw value in the service (urban poor).<br>• CHW paid travel costs.<br>• CHW Willingness to do home visits.	• Intervention home based.<br>• Training: Counselling that was skill based.<br>• Intervention by peers who were socio-culturally alike.<br>• Defined and limited roles for the CHW.<br>• Health professionals and lay workers collaborated to achieve common goals.	• Training triggered a sense of self efficacy, a sense of being able to perform a meaningful function.<br>• The relatedness between the beneficiary and provider led to both responsiveness and responsibility amongst CHW.<br>• Anticipation of being valued by peers for fulfilling the needs of their community.<br>• Assurance that in case of technical difficulty there would be a professional and a system to fall back on.	Post natal, home based counselling by CHW directed at promoting exclusive breastfeeding achieved more exclusive ($p<0.001$) and longer ($p<0.001$) breastfeeding compared to those mothers receiving only childcare counselling home visits by CHW and those receiving no home visits.

Authors, Intervention Focus and Area	Design				
		Context	Intervention characteristics	Mechanisms triggered	Outcomes/Conclusions
Morrow et al, 1999, Mexico City	RCT<br>N=130	• Periurban setting.<br>• The Institute National de la Nutrition has much respect in the area and the study population/community trusts them.<br>• Breastfeeding valued socially.<br>• The BFHI – The Baby Friendly Hospital Initiative was being rolled out at around the same time.<br>• Background of CHW - those who are similar, respected, trusted and in control of their own lives	• Intervention home based and accessible.<br>• Training: Both information and skills (Authors call it "problem solving support to mothers"). Use of visual aids. Practice sessions for 6 months. Intervention by peers with a similar background.<br>• Repetition of intervention helps restore and reinforce behavior.<br>• Long duration of contact with beneficiary.<br>• Timing of intervention (Both intrapartum and postpartum).	• Training triggered a sense of self efficacy, a sense of being able to solve problems.<br>• The relatedness between the beneficiary and CHW led to both responsiveness and responsibility amongst CHW.<br>• CHW profile was meant to trigger an aspirational adoption of the messages by beneficiaries.	Early and repeated contact (counseling) with CHW was associated with a significant increase in exclusivity ($p<0.001$) and duration ($p=0.02$) of breastfeeding (primary outcome) and a reduction in the risk of diarrhoea, compared to control group.
Bhandari et al, 2003, India	RCT<br>N=1115	• Rural settings with an established primary care service.<br>• Breastfeeding is a social norm.<br>• Change throughout the system.<br>• CHW integrated into existing health services.<br>• Community wide engagement through neighbourhood meetings.<br>• Contact with the study team could have motivated the intervention arm to perform better.<br>• Intervention complemented by reiteration of messages from multiple sources, at multiple occasions, at multiple contacts at multiple levels in the local health service.	• Training on task specific knowledge, communication skills, use of tools (charts, visuals aids), focus on detecting and solving problems and skills building (hands on training).<br>• Repetition and routine discussion about the messages at regular intervals by supervisors.<br>• Intervention integrated into the health system.	• Training gave knowledge and triggered a sense of self efficacy; the tools, the skills building and practice helped CHW gain enactive mastery on the tasks and triggered a sense of confidence in being able to solve problems.<br>• An expectation of appreciation by authority (Contact with study team during study period) and possibility of being rewarded may have motivated CHW to perform better.	CHW intervention can achieve exclusive breast feeding ($p<0.0001$), can also reduce the risk of diarrhoea ($p=0.028$).

Authors, Intervention Focus and Area	Design				
		Context	Intervention characteristics	Mechanisms triggered	Outcomes/Conclusions
Coutinho et al, 2005, Brasil	RCT<br>N=350	• Urban settings<br>• Beneficiaries from low socioeconomic strata.<br>• Well established health services (BFHI).<br>• Combination of systems. BFHI at hospital level supported and complemented by community level initiative.<br>• CHW had access to support from hospital nurses and doctors when problems could not be addressed at CHW level.	• Training of CHW was in-depth and included practice sessions.<br>• Teaching aids like illustrated booklets were made available to CHWs.<br>• CHW also addressed other discussions relevant to the infants' age (they were trained to address these questions).<br>• CHWs engaged family level influencers in supporting the new mothers.<br>• Longer contacts initially.<br>• Frequent contacts initially (1st month)	• Training gave knowledge and triggered a sense of self efficacy by addressing the innate need for competence; the tools, the skills building and practice helped CHW gain enactive mastery on the tasks and triggered a sense of confidence in being able to solve problems; the training on other issues related to child development and contact with other family members in addition to above triggered a sense of autonomy and a sense of contribution to the community they served and belonged to.<br>• Confidence, both for themselves and within the community they serve, that personal and material resources are available and accessible in times of need (a back-up BFHI enabled health system).	Improved and sustained breastfeeding practices can be achieved ($p<0.0001$) through a combination of community and hospital based systems and services. This ensures that mothers can access services at home or in their community, especially in the early period after birth.

**Authors, Intervention Focus and Area**	**Design**				
		**Context**	**Intervention characteristics**	**Mechanisms triggered**	**Outcomes/Conclusions**
Haider et al, 2000, Bangladesh	CRCT N=40 zones (726 mother-child pairs)	• Urban population with high level of home deliveries.<br>• Well established health services.<br>• Referral support available (and was reported as being important by CHWs).<br>• There were inconsistencies in what the health staff said about prelacteal feeds (they wrongly promoted it) and what the CHW told the mothers (exclusive breastfeeding).	• Training that was both knowledge and skills based (including interpersonal skills, communication skills, demonstrations, role plays).<br>• Training was followed by practice runs with an accompanying mentor.<br>• Supported by a supervisor (availability of supervisor helped CHWs feel confident and credible; it also helped increase the confidence of the beneficiaries in CHWs).<br>• CHWs made the mothers feel cared for.<br>• CHW were similar to beneficiaries and lived in intervention area.<br>• Willingness to help others was a selection criteria.<br>• Home based service delivery, addressing issues beyond just breastfeeding.<br>• Key family members included in each counseling sessions.	• Individuals with a more favorable apriori attitude towards the job would perform better.<br>• Individuals are motivated when they expect that their work will yield desired outcomes.<br>• Congruence of motives between the individual and the system.<br>• Confidence and a sense of credibility of being a part of the system (intrinsic motivation elicited by external factors), both for CHW and community.<br>• The relatedness between the beneficiary and provider led to both responsiveness and responsibility amongst CHW.<br>• Anticipation of being valued by peers for fulfilling the needs of their community.	Peer counselors can effectively increase the initiation and duration of exclusive breastfeeding ($p<0.0001$).

Authors, Intervention Focus and Area	Design				
		Context	Intervention characteristics	Mechanisms triggered	Outcomes/Conclusions
Leite et al, 2005, Brazil	RCT<br>N=1003	• Urban setting.<br>• Poor beneficiary population<br>• Hospital settings.<br>• State already has a network of CHW, beneficiaries familiar with the concept.<br>• CHW had back up support from professionals (referral).<br>• BFHI has been implemented in the rest of the system.	• Training: A theory-practice course; knowledge and skills focused.<br>• Intervention focused on listening.<br>• Intervention targeting the creation of a social support network.<br>• Home based delivery of services.	• Training triggered a sense of self efficacy, a sense of being able to solve problems	Breastfeeding practices can be improved with a home visit programme carried out by lay counselors. Intervention was more effective in delaying the abandonment (p=0.00002) of breastfeeding than maintaining the children on exclusive breastfeeding (p=0.04).
**Malaria treatment**					
Kidane et al, 2000, Ethiopia	CRCT<br>N=37 Villages	• Rural setting.<br>• Strong community solidarity (in the context of collective adverse experiences and environment).<br>• CHW supported by local public health services (training, receiving referrals, supervision and logistic support).<br>• Disciplined beneficiary population.<br>• No other income source for the CHW.	• Community / Home based intervention.<br>• CHW trained on symptom recognition, recognition of side effects and treatment.<br>• Election / selection of CHW by the beneficiaries themselves.<br>• CHW were accessible (within the locality).<br>• Provision of pictoral tools (job-aids) to facilitate treatment decisions.<br>• Intensive community engagement and consultation.<br>.	• Realization of being valuable to one's community and<br>• Perception of improvement in social status.<br>• Sense of responsibility to the community.<br>• Confidence in self and a sense of credibility of being a part of the system (intrinsic motivation and performance facilitated by external factors).	A major reduction (P<0.003) in under-5 mortality can be achieved in holoendemic malaria areas through training local mother coordinators (CHW) to teach mothers to give under-5 children antimalarial drugs when required.

Authors, Intervention Focus and Area	Design				
		Context	Intervention characteristics	Mechanisms triggered	Outcomes/Conclusions
**Diarrhoea prevention**					
Luby et al, 2006, Pakistan	CRCT<br>N= 47 neighbourhoods with 1340 households	• Urban squatter colony<br>• Low socioeconomic settings.<br>• Poor water and sanitation infrastructure.<br>• Poor access to health services.<br>• Poor neighbourhood which valued the material being given (authors talk of possible courtesy bias in reporting of diarrhoea episodes by households).<br>• Community engagement, community level meetings and discussions.<br>• Involvement of community elders.<br>• Referral support for those identified with diarrhoea etc.<br>• The organization (HOPE) has worked in the area for long. Is a trusted and credible provider.	• Home visits by CHW<br>• Intensive intervention – weekly visits by CHW.<br>• Training of CHW included specific activities that were to be taught to beneficiaries (Knowledge + specific skills were imparted).<br>• Use of tools like slide shows, video tapes, pamphlets.<br>• Rigorous supervision.	• An anticipation that one would be held accountable for a certain outcome.<br>• A realization that being a part of an organization with a history of credible services, one would be expected to deliver.<br>• Realization that the service is valued by the community.	A CHW intervention promoting hand washing and drinking water treatment significantly reduced diarrhoea separately. There was no benefit by combining hand washing promotion with water treatment.

Authors, Intervention Focus and Area	Design				
		Context	Intervention characteristics	Mechanisms triggered	Outcomes/Conclusions
			**Nutrition**		
Schroeder et al, Marsh et al, 2002, Vietnam	CRCT N=12 communes (240 children)	• Rural setting.<br>• Poor beneficiaries with bad nutrition profile<br>• Intervention part of the public health system.<br>• Good access to referral services.<br>• Enthusiastic beneficiaries.<br>• Intervention contaminated by other agents giving nutritional supplements.<br>• Concomitant implementation of a detergents initiative contaminated the intervention.<br>• A Universal de-worming campaign diluted the effect.<br>• Baseline nutrition status of population was good, very few were severely malnourished.	• Home visits.<br>• Training – task focused, skills based, learning by doing.<br>• Training content (Knowledge) based on local knowledge (gathered through positive deviant interviews). Beneficiaries could relate to it.<br>• Intensive support for those in need.<br>• Group learning sessions.	• Individuals with a favorable apriori attitude towards the job would perform better.<br>• Motivation because of the expectation that their work will yield desired outcomes and would be valued.<br>• The relatedness between the beneficiary and provider begeted both responsiveness and responsibility amongst CHW.<br>• Assurance that in case of technical difficulty there would be a professional and a system to fall back on.	Children in the community based integrated nutrition intervention did not show statistically significant better growth than comparison children.

Authors, Intervention Focus and Area	Design				
		Context	Intervention characteristics	Mechanisms triggered	Outcomes/Conclusions
**IMCI**					
Pence et al, 2005, North Ghana	CRCT<br>N=4 Areas (53134 children)	• Rural setting<br>• Poor beneficiaries.<br>• Poor infrastructure (water, sanitation).<br>• High infant and child mortality.<br>• Established primary care infrastructure.<br>• CHW probably part of the political establishment, rather than the local public health service.<br>• CHW appointed by the Village Development Committee, probably not selected or elected by community.	• Intervention designed to harness cultural resources such as chieftaincy, lineage, and social network systems.<br>• Training for six weeks followed by quarterly refreshers.<br>• Roles too broad.<br>• Responsibilities not well defined.<br>• Authors conclude: A limited and well defined role (limited to education and outreach coordination and family planning) would be more beneficial.<br>• CHW roles and limitations not clear to the community.<br>• CHWs perceived as replacements for the professional health worker (causing delays in seeking treatment).	• Poor performance begeted by poor initial outcomes.<br>• Lack of congruence between the goals of CHW and health professionals led to a negative feedback compromising credibility (of CHW) and driving poor performance.<br>• Uncertainty because of ill defined roles led to lowered motivation, less involvement (both at CHW and beneficiary level) and poor performance.<br>• Lowered sense of self efficacy and lack of relatedness with the beneficiary and health system compromised the motivation and performance.	CHW intervention associated with an 11 percent increase in mortality, primarily driven by a 124 percent increase in early child mortality. In a comparison area, under-five mortality fell by 5 percent during the same period and fell by 4.5 deaths per 1000 per year in areas served by more accessible professional medical personnel. Results raise questions about the benefits to children's survival of relying on CHW.
